# Chronic traumatic encephalopathy—a blueprint for the bridge between neurological and psychiatric disorders

**DOI:** 10.1038/s41398-020-01111-x

**Published:** 2020-12-08

**Authors:** Onder Albayram, Sait Albayram, Rebekkah Mannix

**Affiliations:** 1 Division of Cardiology, Department of Medicine, Medical University of South Carolina, Charleston, SC 29425 USA; 2 Department of Neuroscience, Medical University of South Carolina, Charleston, SC 29425 USA; 3 Department of Radiology, University of Florida College of Medicine, Gainesville, FL 32610 USA; 4 Division of Emergency Medicine, Boston Children’s Hospital, Harvard Medical School, Boston, MA 02115 USA

**Keywords:** Psychiatric disorders, Scientific community

## Abstract

Chronic traumatic encephalopathy (CTE) is a perplexing condition characterized by a broad and diverse range of neuropathology and psychopathology. While there are no agreed upon or validated clinical criteria for CTE, case series of CTE have described a wide range of neuropsychiatric symptoms that have been attributed to repetitive traumatic brain injuries (rTBI). However, the direct links between the psychopathology of psychiatric and neurological conditions from rTBI to CTE remains poorly understood. Prior studies suggest that repetitive cerebral injuries are associated with damage to neural circuitry involved in emotional and memory processes, but these studies do not offer longitudinal assessments that prove causation. More recent studies on novel targets, such as transmission of misfolded proteins, as well as newly advanced non-invasive imaging techniques may offer more direct evidence of the pathogenesis of CTE by tracing the progression of pathology and display of related behavioral impairments. Understanding this interface in the context of rTBI can play an important role in future approaches to the definition, assessment, prevention, and treatment of CTE and mental illnesses.

## Introduction

Repetitive traumatic brain injury (rTBI), often associated with concussions in contact sports and blast injuries in military personnel, is a legitimate public health concern^1–3^. Populations at high risk for rTBI are thought to be at risk of chronic traumatic encephalopathy (CTE), known also as dementia pugilistica or punch-drunk syndrome, which manifests as a composite syndrome of mood disorders, neuropsychiatric disturbance, and cognitive impairment^4–7^. The evidence linking CTE pathology to neuropsychiatric manifestations is indirect and controversial. Since CTE is currently an autopsy confirmed diagnosis, no prospective studies have demonstrated the progression of CTE pathology with worsening neuropsychiatric symptomatology^8^. However, indirect evidence suggests that head trauma may play a causative role in the development of neuropsychiatric symptomatology^9–11^. Several studies have suggested that the early-stage and mid-stage of repetitive-mild/moderate cerebral injury closely mimic the symptoms of psychological disorders associated with a clear pattern of neuro-psychopathology^12,13^, though other studies dispute whether these findings can be definitively attributed to head trauma^14^. Retrospective case studies have manifested that neuropsychiatric symptoms are distinct features in the stages of CTE^15,16^, prior to be diagnosed with neurological deficits, mimicking those seen in psychiatric disorders^17^. On the other hand, psychotropic medications (e.g., antidepressants, mood stabilizers, and antipsychotics) were often prescribed to patients ultimately diagnosed with acute and chronic TBI^18–20^.

In addition to clinical symptomatology, structural considerations may further support the link between rTBI and CTE clinical symptomatology^21^. For example, cerebral injuries that result from repetitive head trauma may target brain circuits that are associated with both neurological and psychiatric symptoms^22–26^. Early retrospective studies suggest that the pathogenesis of rTBI may not occur at random, but rather it follows characteristic pathways, which follow the neural circuit primarily involved in episodic memory (mesial temporal, posterior cingulate/precuneus, lateral temporoparietal)^15,27,28^.

Actually, the fundamental mechanisms of developing post-TBI symptoms likely arise as a result of weakened function and activation of brain circuitry that transpires in maladaptive responses. As a result of this viewpoint, the theme of brain circuit disruption contributes to psychological abnormalities for many of the observed post-TBI symptoms. Indeed, the development of CTE can also be affected by a number of pre-injury conditions, which may affect a recovery and prognosis after injury. These include, but are not limited to, mental and physical stabilities, social class, gender, race, nutrition, and alcohol or substance abuse^29–31^. Indeed, the variation in the pathogenesis of brain trauma by interrupting brain circuits highlights the relevance of studying the progression of injury. The white matter degeneration developing from diffuse axonal injury has the potential to detach and shear neuronal circuits between various brain regions, with implications for both animal and patient behavioral disorders. On the other hand, the etiology of CTE is currently limited to post-mortem cross-sectional assessments rather than the more desirable protocol of prospective longitudinal studies.

This situation highlights the probability that some cases of repetitive head injury may not be progressive but are instead a constant disease state with unpredictable levels of severity, as revealed through autopsy. This probably represents distinct reactivity to repetitive brain injury and/or the age or period of time between experiencing head trauma and death. Indeed, the incidence of pathological changes in the lack of symptoms is unknown, as are the causes controlling the connection between neuropathological load and clinical verge. However, it can be difficult to set direct and reliable relations between an injury, the neural network fluctuations, and the corresponding behavioral discrepancy due to the apparent heterogeneity of initial head impact and its pathological pattern. It has been shown that some of these long-term consequences are mediated with maladaptive changes in brain networks.

For example, amplified stimulation in the default state network is associated with impairments of sustained attention after brain trauma. Furthermore, changes in functional brain connectivity are anticipated to elucidate sensorimotor deficits after mild brain injury^32^. In addition, such changes can also differentially address brain regions associated with episodic memory^33^, as well as target particular brain circuits related to cognitive function^34^ and procession sensory information^35^. Synchronization is essential not just for characterizing intrinsic neuronal networks but is also crucial at the cellular scale for assisting interaction, as it momentarily binds neurons together into functional ensembles^36,37^. Similarly, memory processing is mainly contingent upon coherence, which allows long-distance interaction between various brain regions^38,39^. Recent findings in human imaging studies identified that TBI disturbs synchronization^40–42^, leading to the probable increase or decrease in functional network connectivity that contributes to long-term cognitive defects.

The use of newly advanced non-invasive imaging techniques for imaging areas of cerebral injuries, such as, structural magnetic resonance imaging (MRI), susceptibility-weighted imaging (SWI), diffusion MRI, functional magnetic resonance imaging (FMRI), single-photon emission computed tomography perfusion abnormalities (SPECT) positron-emission tomography (PET) imaging from tau-binding, glucose-binding, and GABA receptor-binding radionuclides and magnetic resonance spectroscopy (MRS) in parallel with psychological inventories of emotional symptoms, have enormous potential^43,44^. These imaging techniques have great potential to precisely characterize the patterns of injuries and corresponding behavior, and to ultimately allow for the further study of intrinsic cerebral connectivity. Early and late disease tracing may provide an ideal model system for understanding the network and brain dynamics associated with the key symptoms of mental illness. Such a discovery would be a critical step towards effective treatment of psychiatric and neurological disorders.

Indeed, structural imaging that can highlight the behavioral circuit could help pinpoint the neuropsychiatric symptoms used in the diagnostic criteria for Traumatic Encephalopathy Syndrome (TES). However, the complementary features in TES and its psychiatric outcomes can be complicated. Psychiatric manifestations can be ephemeral or perpetual. Biological variables (type of injury/duration, co-morbidities, family/past history of neuropsychiatric disorders, drug of abuse, the social and cultural factors) need to be contemplated in the formulation.

## Behavioral features from TBI to CTE

The neuropsychiatric manifestations attributed to CTE are manifold and diverse, and include cognitive processes (e.g., memory, visuospatial abilities, executive control), motor coordination and psychological symptoms (impulsivity, depression, emotional instability, substance abuse, and suicidal thoughts, amongst others)^45–47^. Although CTE is portrayed as a single disease, a spectrum of CTE severity is described in the literature; investigators have attempted to correlate clinical symptoms to neuropathology for each of the four proposed stages of CTE^15^. It remains to be seen whether such diagnostic criteria could be used to diagnose and manage disease in living patients. Importantly, many of the neuropsychiatric features of CTE are also reported in the broader TBI literature, suggesting common neuropathologic mechanisms could be in play.

Populations at high risk for cerebral brain injuries show high incidences of neuropsychiatric sequelae^48,49^. Major depression is one of the most prevalent psychiatric abnormalities observed in individuals after TBI. Earlier studies showed that brain imaging soon after head trauma shows left dorsolateral frontal lesions and/or left basal ganglia lesions, which are key risk factors for major depression in TBI^50^. The depressive phenotype is defined by persistent sadness, inability to feel pleasure, poor sleep, feelings of guilt or low self-worth, and, at times, suicide^50–53^. In addition, TBI often leads to impaired social functioning in domains such as interpersonal interactions and relationships, community, and civic life^54,55^. In contrast, manic episodes, symptoms of impatience, increased activity, impulsivity, and at times delusions or hallucinations, are far less common than major depression^49,56,57^.

Anxiety disorders are also typical in post-TBI and CTE patients and include posttraumatic stress disorder, obsessive-compulsive disorder, and generalized anxiety disorder^15,46,58–60^. Chronic anxiety has been specifically associated with brain trauma of the right hemispheric cortical lesions^61^. Another predominant TBI symptom is apathy, defined by indifference in daily life, decreased drive and motivation, lack of emotion, and decreased socialization^62,63^. Although the above description may seem indicative of depression, apathy often manifests itself as a distinct symptom^64^. Interestingly, some research supports that apathy stems from injury of the mesial frontal lobe and subcortical structures^65,66^. TBI has been also strongly associated with sleep disruption^67,68^ which might be due to the neurological damage of the glymphatic system^69,70^.

One caveat to these behavioral symptomologies of TBI is that pre-existing psychological conditions can grossly affect the diagnosis and treatment outcome of patients suffering from cerebral brain injuries and thus, obscure the connection between injury and disease progression^61,71,72^. Such issues that arise from this condition include, premorbid states of alcohol dependence, impulsive behavior, lack of social support, and other psychiatric disturbances^73,74^. Indeed, there might a complex variation between the patients’ distinct pattern of brain connectivity and genetic variants in the development of CTE, and each individual reveal different kind susceptibility to specific types of psychiatric disorder during the progression of disease^74–78^.

On the other hand, there are still significant questions concerning symptom onset, course, and etiologic factors owing to the lack of longitudinal data. Most research correlating repetitive brain injury and clinical outcomes relies on the self-report of older former athletes and has led to the assumption that a clinically silent period follows repetitive head trauma. However, major lifestyle adjustments and transitions accompanying retirement from sport can strongly influence the behavioral and mood symptoms that are considered to be earlier clinical symptoms of CTE. This is a critical issue in correctly defining reference groups for normative comparisons. Indeed, it is hypothesized that repetitive brain injury exposure interacts with other genomic and environmental factors to modify susceptibility to long-term behavioral dysfunction. A retrospective clinicopathological study concluded that CTE patients who originally manifested with behavioral symptoms commonly had substance abuse issues, family history of psychiatric disorders, and suicidal thoughts^45^. In order to concretely differentiate between pre-TBI and post-TBI, novel strategies must be implemented.

## Pathological features from TBI to CTE

The neural mechanisms associated with rTBI and CTE might be a promising model for uncovering the neural networks of psychopathology and solidifying the relationship between the brain and behavior. Anatomically, current evidence suggests that CTE does not spread from the initial injury in a random manner, but rather it propagates along neural networks that are both structurally and functionally linked^15,79^. While a direct and clear pathophysiological link between rTBI and the evolution of CTE has not been found, the first NINDS/NIBIB consensus meeting has helped elucidate this relationship by further establishes a neuropathological standard for distinguishing CTE brains from other neurodegenerative brain disease^79^.

In detail, retrospective postmortem studies with patients that had a history of repetitive head injury showed neuropathological changes within the brain involving tau pathology, TAR-DNA binding protein 43 (TDP-43) proteinopathy, neuroinflammation, microvascular injury and disruption of the blood brain barrier^15,80,81^. These pathological events may be affected by both the severity and the duration of head injury^82^. In the early stage of the CTE brains, retrospective studies show that tau aggregates are primarily detected in subcortical regions, including frontal, temporal, and parietal cortices^79^. However, during the later stages of progression, tau aggregates are more widely spread throughout the cerebral cortex, medial temporal lobe, amygdala, hypothalamus, thalamus, and brain stem^15,16,45^. This pattern classifies CTE from other tauopathies, such as AD and frontotemporal dementia^79^. On the other hand, different from AD, CTE typically involves neurotic amyloid-beta aggregates only in advanced stages of disease^83^.

Retrospective studies show that in the later stages of CTE, definitive macroscopic changes illustrate a significant reduction in brain weight, pronounced degeneration of the thalamic nuclei and mammillary bodies, as well as significant atrophy prominent in the frontal and temporal cortices and medial temporal lobe^15,79–84^. Thinning of the amygdala, locus coeruleus and substantia nigra were also noted as hallmarks of advanced-stage CTE^7^. However, because the medial structures of the limbic system, including the amygdala, are commonly associated with the greatest atrophy in CTE^15,16,46^, a number of studies are venturing to link these anatomical changes to symptoms, such as the link between amygdala structural volume loss and suicidal ideation^85^.

Retrospective case studies have reported clear neuroinflammatory activation in the brains of former football players who had more than one injury in brain regions such as the hippocampus, entorhinal cortex, para hippocampal cortex, supramarginal gyrus, amygdala, and temporal pole^86,87^. A recent retrospective study revealed that repetitive head injuries are associated with chronic microglial activation, which may moderately mediate the consequence of repetitive injuries on the development of neurofibrillary tangles and dementia in CTE^86^. Evidence has increasingly shown that chronic neuroinflammation can trigger various neuropathological changes, including neuronal apoptosis and hyperphosphorylation of tau aggregates. Besides, neuroinflammation may also play a key role in the increased risk for the development of neurodegenerative diseases for those with a history of TBI. A number of studies also showed that the persistent focal traumatic microvascular injury, where perivascular astrocytes and microglia join, might be an important contributing factor through evolving pathways into CTE^15,16,87,88^.

Indeed, there is a large gap in our understanding of how tau, vital for the physiologic function in healthy neurons, can become pathogenic and lead to behavioral dysfunction in the setting of TBI and/or CTE. Populations at high risk for TBI show high incidences of behavioral changes resulting from CTE, but no tau tangle pathology is detected in acute or subacute TBI brains, making the connection between neuronal injury and tangle formation unclear. Interestingly, hyperphosphorylated TDP-43 has been recognized as an early common pathological feature linking frontotemporal dementia (FTD), amyotrophic lateral sclerosis (ALS) and Alzheimer’s disease (AD)^89^. It has also been detected in more than 80% CTE cases^90^, raising the question of whether there is a pathological synergy between TDP-43 and tau proteinopathies in CTE. Furthermore, preclinical studies showed that caspase-cleavage of TDP-43 caused by TBI leads directly to redistributed TDP-43^79^. Thus, TDP-43 pathology can be considered both a primary and secondary feature of CTE.

Not every individual who sustains repetitive head injuries will exhibit the neuropathology characteristic of CTE, which adds another layer of complexity in linking neuropathology and psychiatry^91^. One theory as to why some patients are more or less susceptible to CTE following rTBI is due to differences found in the cognitive reserve. The affect that age of symptom onset results from cognitive reserve, which is the mind’s resistance to developing symptoms following brain damage. Cognitive reserve is a multifaceted symptom modifier and has both genetic and environmental components, but researchers found that occupational achievement was the best indicator of the age of disease onset^92^.

Overall, the relationship between rTBI, CTE and focal brain lesions on neural activity presents an ideal model for understanding the behavioral changes associated with specific damaged brain circuits. Interestingly, in TBI, varying amounts of pathology are seen within the cingulum bundle, a tract that includes connections between the posterior cingulate cortex and ventromedial prefrontal cortex^93^. This damage correlates with impairments in sustained attention, whereby patients are unable to maintain attentional focus over extended periods of time^94,95^. Increased damage to the cingulum bundle is associated with worsening attention, a deficit that is also predicted by reductions in functional connectivity within the default mode network^96^. In order to fully understand these neurodegenerative processes, we must first accurately visualize and trace the path along which cellular damage occurs, as well as the mode of action by which this degeneration happens. At this point, long-term longitudinal studies on the order of the Framingham or Honolulu Heart Study are warranted to verify the serial assessments from the onset of repetitive head trauma through midlife and then into later life, when symptoms emerge in a significant percentage of cases. Indeed, it may also to help clarify whether immediate vs. later clinical manifestations represent different disease mechanisms or subtypes through detailed tracking of symptom type, onset, and fluctuations, starting at the point when exposure to repetitive brain trauma ceases.

## Relevance of pre-clinical models of TBI to human CTE

With the increased awareness of the link between CTE and TBI, a significant number of studies have begun to explore the pathophysiological and behavioral mechanisms relevant to CTE using animal models. Indeed, producing an clinically relevant animal model of TBI is difficult due to a number of variables, such as the complexity of scaling the equivalent force of head trauma seen in humans onto a much smaller brain, the time between injuries, and how many injuries occur over the animal’s lifespan^97,98^. There are numerous animal models of TBI each of which model different aspects of clinical TBI pathology and behaviors. Some of the most widely published models include closed head weight drop, the controlled cortical impact (CCI) and fluid percussion (FP) injuries^98^. However, it is important to acknowledge that TBI is a highly heterogeneous condition that can result in a broad range of pathological changes that may manifest in different behavioral dysfunction.

CCI utilizes a rigid impactor to deliver mechanical energy to the dura of the brain of an animal restrained in a stereotaxic device, producing a focal contusive injury^99^. Importantly, the use of the restraint does not allow for linear or rotational acceleration forces that are commonly seen in human concussive injuries^100,101^. In the lateral FP (LFP) model, injury is induced by administering a craniotomy to the animal and applying a fluid pressure pulse to the intact dura of the brain, thus producing displacement and deformation of neural tissue^102^. Like the CCI model, this injury does not produce the linear and rotational forces seen in clinical concussive injuries^103,104^, and the need for a craniotomy to perform the injury can introduce complications such as infection^105,106^. In the closed head weight drop model, a weight is dropped directly onto the intact skull of the animal, causing movement of the unrestricted head^107^. This model results in neurobehavioral changes associated with concussive injuries similar to the others while also generating additional linear and rotational forces to mimic human TBI^108,109^. Differences often occur in the duration and time between impacts across different models^110^. Importantly, many models also observe the long-term effects post injury, an important factor in trying to replicate CTE as a neuropathology^111–113^. Because of the similarity in the results of these established models, more research is needed to determine what factors induce pathological changes, including the type of mechanical force, injury frequency, and severity of injury.

It is worth noting that there is still a lack of understanding of the fundamental link between pathological mechanisms and the different neurobehavioral changes that occur in TBI, making it difficult to trace the mechanisms to the neurobehavioral alterations that occur after TBI in both patients and rodents^114^. Future studies are necessary to clarify this and further corroborate the relevance of neurobehavioral sequels in animal models. Because several of the preclinical models’ available impact the same limbic regions, they could be used as translational implements to study pathophysiology associated with mental disorders. For example, many reports describe an increase in depressive-like behavior in models of TBI, in good accordance with the clinical studies. The most convincing hallmark of those reports is the concurrence of the depressive-like phenotype, which is recognized despite the fact that these studies are being handled in different institutes using a wide range of parameters, such as age, animal species, injury models, severity of injury and time post-injury^100,101,114–119^. Therefore, these findings suggest that depression is a robust consequence of TBI.

Regardless of their limitations, animal models of concussion are an important factor that allows us to develop an insight into the long-term effects of repeated head impacts. In addition, animal models of repeated concussion should also be highly reflective of the current descriptions of CTE, leading to progressive cognitive deficits, mood changes, and the gradual appearance of key neuropathological features such as neurofibrillary tangles, deposits of phosphorylated TDP-43, changes in white matter integrity, and sustained microglial activation.

## Conclusion

Unlocking the intersections between the psychiatric and the neurological symptoms of rTBI and CTE will allow us to use these disorders as prototypes in understanding other mental illnesses. This will ultimately prevent the onset of the psychological and neurological damage associated with the onset of chronic neurodegeneration. Evolving science on the structure and function of connectivity between regions of the brain, along with the recognition of distinct and predictable patterns of degeneration associated with traumatic cerebral injury and CTE, will make it possible to study how other pathologies, specifically psychopathologies, function within the same, or similar, pathways. We are currently limited in our capacity to address many psychiatric disorders due to the inability to relate any significant structural connectivity changes to the behavioral changes’ characteristic of known disorders. However, the model systems presented in TBI have great potential to aid in the discovery of more effective and targeted methods for the assessment and treatment of these psychiatric disorders. Early pathological markers may be at the foundation of this approach, as it has been shown to provide accurate temporal and spatial tracking of pathological progression and can shed light on the exact neural circuitry involved with the aforementioned psycho and neuro-pathologies.
